# Cluster of Carbapenemase-Producing Carbapenem-Resistant *Pseudomonas aeruginosa* Among Patients in an Adult Intensive Care Unit — Idaho, 2021–2022

**DOI:** 10.15585/mmwr.mm7231a2

**Published:** 2023-08-04

**Authors:** Megan E. Cahill, Martha Jaworski, Victoria Harcy, Erin Young, D. Cal Ham, Paige Gable, Kris K. Carter

**Affiliations:** ^1^Epidemic Intelligence Service, CDC; ^2^Idaho Division of Public Health; ^3^Utah Public Health Laboratory; ^4^Division of Healthcare Quality Promotion, National Center for Emerging and Zoonotic Infectious Diseases, CDC; ^5^Division of State and Local Readiness, Center for Preparedness and Response, CDC.

**Summary**

**What is already known about this topic?**

Treatment of carbapenemase-producing carbapenem-resistant *Pseudomonas aeruginosa* (CP-CRPA) infections is challenging because of antibiotic resistance. CP-CRPA infections are highly transmissible in health care settings because they can spread from person to person and from environmental sources.

**What is added by this report?**

CP-CRPA was detected in two patients who each spent approximately 1 month in the same intensive care unit (ICU) room, 4 months apart. Isolates from both patients contained a carbapenemase-producing gene. The same gene type was also detected in isolates from one of the ICU room sinks. Control measures included discontinuing room use pending sink drain biofilm disinfection.

**What are implications for public health practice?**

Multifaceted interventions, including sink hygiene practices, engineering controls, and administrative controls, are critical to limiting multidrug-resistant organism spread in health care settings.

## Abstract

Treatment of carbapenemase-producing carbapenem-resistant *Pseudomonas aeruginosa* (CP-CRPA) infections is challenging because of antibiotic resistance. CP-CRPA infections are highly transmissible in health care settings because they can spread from person to person and from environmental sources such as sink drains and toilets. During September 2021–January 2022, an Idaho hospital (hospital A) isolated CP-CRPA from sputum of two patients who stayed in the same intensive care unit (ICU) room (room X), 4 months apart. Both isolates had active-on-imipenem metallo-beta-lactamase (IMP) carbapenemase gene type 84 (*bla*_IMP-84_) and were characterized as multilocus sequence type 235 (ST235). A health care–associated infections team from the Idaho Division of Public Health visited hospital A during March 21–22, 2022, to discuss the cluster investigation with hospital A staff members and to collect environmental samples. CP-CRPA ST235 with *bla*_IMP-84_ was isolated from swab samples of one sink in room X, suggesting it was the likely environmental source of transmission. Recommended prevention and control measures included application of drain biofilm disinfectant, screening of future patients who stay in room X (e.g., the next 10 occupants) upon reopening, and continuing submission of carbapenem-resistant *P. aeruginosa* (CRPA) isolates to public health laboratories. Repeat environmental sampling did not detect any CRPA. As of December 2022, no additional CP-CRPA isolates had been reported by hospital A. Collaboration between health care facilities and public health agencies, including testing of CRPA isolates for carbapenemase genes and implementation of sink hygiene interventions, was critical in the identification of and response to this CP-CRPA cluster in a health care setting.

## Investigation and Results

A collaborative investigation involving the Idaho Division of Public Health (IDPH) Healthcare Associated Infections (HAI) program, Idaho Bureau of Laboratories (IBL), the Utah Public Health Laboratory (UPHL), and CDC was undertaken to identify the etiology of the infection. This activity was reviewed by CDC and was conducted consistent with applicable federal law and CDC policy.*

On September 17, 2021, an Idaho hospital (hospital A) collected sputum by endotracheal tube aspiration of a woman aged 50–65 years (patient 1), who received mechanical ventilation during 3 of 5 weeks of hospitalization in an intensive care unit (ICU) room (room X). Carbapenemase-producing carbapenem-resistant *Pseudomonas aeruginosa* (CP-CRPA) was detected only in this fifth serial sputum specimen, suggesting hospital-acquired infection.

Carbapenem-resistant *P. aeruginosa* (CRPA) isolates in Idaho are voluntarily submitted to IBL for detection of carbapenemase genes.^†^ IBL detected phenotypic carbapenemase production using the modified carbapenem inactivation method, but did not detect any of the four most common carbapenemase genes^§^ using real-time polymerase chain reaction (PCR), which suggested that a different carbapenemase gene was present. Whole genome sequencing by UPHL, a regional laboratory in CDC's Antibiotic Resistance Laboratory Network, detected active-on-imipenem metallo-beta-lactamase (IMP) carbapenemase gene type 84 (*bla*_IMP-84_) and characterized the isolate as multilocus sequence type 235 (ST235). IMP is one of the less commonly reported carbapenemase genes, all of which encode for enzymes that degrade carbapenems and other β-lactam antibiotics and are associated with multidrug-resistant phenotypes (*1*,*2*). The IDPH HAI program provided guidance to hospital A, including recommending submitting all CP-CRPA samples for testing.

On January 25, 2022, hospital A collected a third sputum specimen from a woman aged >65 years (patient 2), who occupied room X while receiving mechanical ventilation for 4 weeks. CP-CRPA was isolated only from this final serial specimen, suggesting hospital-acquired infection. On January 26, patient 2 was transferred to a long-term care facility (hospital B) and not placed on contact precautions during her 10-day stay. Hospital A sent a contact precaution recommendation based on CP-CRPA detection, but it was not directed to hospital B’s infection preventionist.

No patients were placed in room X after the second clinical CP-CRPA isolate was reported. UPHL confirmed that patient 2’s clinical isolate was ST235 with *bla*_IMP-84_, which supported epidemiologic linkage between patients. After the report of a second isolate with ST235 and *bla*_IMP-84_, the IDPH HAI team planned an in-person visit to investigate this cluster associated with room X. During March 21–22, 2022, the team met with hospital A’s infection prevention team to review policies, procedures, and patient histories. Between occupancies of patients 1 and 2, a total of 16 patients occupied room X for a median of 3.5 days (range = 1–12 days). Records showed that at least one respiratory specimen was cultured from each of five patients who occupied room X; however, no CP-CRPA was isolated from these specimens.

During this visit, the IDPH HAI team collected environmental samples based on consultation with CDC. Plumbing was sampled because *P. aeruginosa* persists in biofilm, which is a collection of microorganisms that are adherent to one another and to a surface, such as pipes. Water samples and swabs from two sinks and one toilet were collected from room X. Nondisposable parts of a ventilator used by patient 1 were swabbed. No ventilator used by patient 2 was available for testing. CP-CRPA ST235 with *bla*_IMP-84_ was identified from one sink, including swab samples from the drain, p-trap (a bend in a drain pipe that contains water, which forms a seal to block entry of sewer gases), and counter; IMP+ *P. aeruginosa* was recovered from the p-trap water sample and one of seven toilet bowl water samples. After sequencing, all isolates were uploaded to BioProject PRJNA288601 and analyzed via Pathogen Detection (*3*); cluster PDS000105853.3 included all isolates from this investigation and no others as of May 23, 2023 (Supplementary Figure, https://stacks.cdc.gov/view/cdc/131485) (Supplementary Table 1, https://stacks.cdc.gov/view/cdc/131483). Clinical isolates were most similar to each other and the sink drain isolate with 14 and 16–22 single nucleotide polymorphism differences, respectively (Supplementary Table 2, https://stacks.cdc.gov/view/cdc/131484).

Screening for CP-CRPA colonization by rectal swab (by PCR^¶^ and culture) or sputum sample of two other patients with current or recent stays in hospital A's ICU and 19 patients at hospital B was conducted during the week of March 14, 2022; no CP-CRPA was detected, suggesting no person-to-person transmission despite patient 2 not being on contact precautions at hospital B. 

## Public Health Response

The IDPH HAI team's recommendations to hospital A, in consultation with CDC, included the following: 1) close room X pending sink drain biofilm disinfection with a foam peracid mixture** EPA-registered for drain biofilm disinfection against *P. aeruginosa*, 2) add sink splash guards to reduce counter contamination from the drain, 3) add the disinfectant to weekly cleaning procedures for all ICU room drains, 4) introduce sink hygiene practices^††^ such as designated handwashing sinks, 5) confirm that infection preventionists receive contact precaution recommendations after patient transfers, 6) collect screening specimens from room X occupants (e.g., the next 10 occupants or, if rarely occupied, occupants during a 3-month period) for CP-CRPA detection, and 7) continue submitting CRPA isolates to IBL. The disinfectant product was applied per manufacturer’s instructions during May 9–27, 2022 (daily for 3 days and then every 3–5 days for four additional applications). Thirteen days after the seventh disinfectant application and 11 weeks after the initial visit, the IDPH HAI team collected swabs of the previously contaminated sink bowl and drain; *P. aeruginosa* was not isolated. As of December 2022, hospital A has reported no additional CRPA clinical isolates.

## Discussion

This investigation highlights how collaboration among hospital A's surveillance program, public health HAI programs, and public health laboratories identified a cluster of CP-CRPA with *bla*_IMP-84_ in two hospitalized patients, no evidence of person-to-person transmission, and one sink as the likely environmental source of CP-CRPA in an ICU room. Risk factors for CRPA include hospitalization, especially while receiving mechanical ventilation (*4*). Both patients in this cluster received prolonged mechanical ventilation and had routine serial sputum cultures for surveillance of infection-related ventilator-associated complications.^§§^ Among the 16 patients hospitalized in room X between the occupancies of patient 1 and patient 2, only five had respiratory specimens cultured during hospitalization, and CP-CRPA was not isolated from any specimen; it is possible that the shorter stays (≤12 days) or lack of mechanical ventilation reduced transmission risk.

Whole genome sequencing of isolates strengthened evidence supporting linkage between patients. Because CP-CRPA persists in the environment, particularly in biofilms formed in premise plumbing (*5*), plumbing within room X was sampled; CP-CRPA isolates from samples collected from one sink were genetically similar to the clinical isolates. Addition of the disinfectant to the sink drain cleaning schedule appeared to be successful in eliminating CP-CRPA; however, optimal frequency of drain disinfection for disrupting CP-CRPA biofilm formation remains to be established, with findings from some studies suggesting that repeated application of disinfectant every 3–7 days could be effective in reducing gram-negative bacterial loads (*6*,*7*).

### Limitations

The findings in this report are subject to at least three limitations. First, although no colonization screening detected CRPA, screening was voluntary and limited to patients currently at either hospital A or B, which limited conclusions about the extent of transmission. Second, hypothesized mechanisms of transmission, including splash directly onto patient care items or contamination of health care personnel or visitors during sink use and subsequent transmission to patients, were not assessed. Finally, reporting and submission of CRPA isolates to IBL is voluntary, which limits knowledge regarding CRPA detection in Idaho.

### Implications for Public Health Practice

Collaboration among hospital HAI staff members and public health agencies improved the strength of evidence supporting recommendations in a CP-CRPA HAI cluster investigation. Multifaceted interventions, including sink hygiene practices, engineering controls to minimize splashing, and administrative controls, are critical to limiting further spread of multidrug-resistant organisms in health care settings (*8*).

## Footnotes

* 45 C.F.R. part 46, 21C.F.R. part 56; 42 U.S.C. Sect. 241(d); 5 U.S.C. Sect. 552a; 44 U.S.C. Sect. 3501et seq.

†
https://arpsp.cdc.gov/profile/arln/crpa

§ Four carbapenemase genes were tested by PCR: *Klebsiella pneumoniae* carbapenemase, New Delhi metallo-beta-lactamase, Verona integron-encoded metallo-beta-lactamase, and oxacillinase-48-like beta-lactamase.

¶
https://www.accessdata.fda.gov/cdrh_docs/pdf16/K160901.pdf

**
https://www3.epa.gov/pesticides/chem_search/ppls/001677-00226-20200205.pdf

††
https://www.cdc.gov/hai/prevent/environment/water.html

§§
https://www.cdc.gov/nhsn/pdfs/pscmanual/10-vae_final.pdf

## References

[1] Sheu CC, Chang YT, Lin SY, Chen YH, Hsueh PR. Infections caused by carbapenem-resistant *Enterobacteriaceae*: an update on therapeutic options. Front Microbiol 2019;10:80. 10.3389/fmicb.2019.00080 30761114 PMC6363665

[2] McKenna M. Antibiotic resistance: the last resort. Nature 2013;499:394–6. 10.1038/499394a 23887414

[3] National Library of Medicine. Pathogen detection. Bethesda, MD: National Library of Medicine; National Center for Biotechnology Information; 2016. Accessed April 24, 2023. https://www.ncbi.nlm.nih.gov/pathogens/

[4] Sabour S, Huang JY, Bhatnagar A, Detection and characterization of targeted carbapenem-resistant health care–associated threats: findings from the Antibiotic Resistance Laboratory Network, 2017 to 2019. Antimicrob Agents Chemother 2021;65:e0110521. 10.1128/AAC.01105-21 34570648 PMC8597727

[5] Weingarten RA, Johnson RC, Conlan S, ; NISC Comparative Sequencing Program. Genomic analysis of hospital plumbing reveals diverse reservoir of bacterial plasmids conferring carbapenem resistance. MBio 2018;9:e02011–7. 10.1128/mBio.02011-17 29437920 PMC5801463

[6] Ramos-Castaneda JA, Faron ML, Hyke J, How frequently should sink drains be disinfected? Infect Control Hosp Epidemiol 2020;41:358–60. 10.1017/ice.2019.316 31918767

[7] Jones LD, Mana TSC, Cadnum JL, Effectiveness of foam disinfectants in reducing sink-drain gram-negative bacterial colonization. Infect Control Hosp Epidemiol 2020;41:280–5. 10.1017/ice.2019.325 31801646

[8] Parkes LO, Hota SS. Sink-related outbreaks and mitigation strategies in healthcare facilities. Curr Infect Dis Rep 2018;20:42. 10.1007/s11908-018-0648-3 30128678

